# Dynamic image denoising for voxel-wise quantification with Statistical Parametric Mapping in molecular neuroimaging

**DOI:** 10.1371/journal.pone.0203589

**Published:** 2018-09-05

**Authors:** Stergios Tsartsalis, Benjamin B. Tournier, Christophe E. Graf, Nathalie Ginovart, Vicente Ibáñez, Philippe Millet

**Affiliations:** 1 Division of Adult Psychiatry, Geneva University Hospitals, Geneva, Switzerland; 2 Department of Psychiatry, Faculty of Medicine, University of Geneva, Geneva, Switzerland; 3 Addictology Division, Geneva University Hospitals, Geneva, Switzerland; 4 Division of Medical Rehabilitation, Geneva University Hospitals, Geneva, Switzerland; 5 Clinical Neurophysiology Unit, Division of Psychiatric Specialties, Geneva University Hospitals, Geneva, Switzerland; Universitat de Valencia, SPAIN

## Abstract

**Purpose:**

PET and SPECT voxel kinetics are highly noised. To our knowledge, no study has determined the effect of denoising on the ability to detect differences in binding at the voxel level using Statistical Parametric Mapping (SPM).

**Methods:**

In the present study, groups of subject-images with a 10%- and 20%- difference in binding of [^123^I]iomazenil (IMZ) were simulated. They were denoised with Factor Analysis (FA). Parametric images of binding potential (BP_ND_) were produced with the simplified reference tissue model (SRTM) and the Logan non-invasive graphical analysis (LNIGA) and analyzed using SPM to detect group differences. FA was also applied to [^123^I]IMZ and [^11^C]flumazenil (FMZ) clinical images (n = 4) and the variance of BP_ND_ was evaluated.

**Results:**

Estimations from FA-denoised simulated images provided a more favorable bias-precision profile in SRTM and LNIGA quantification. Simulated differences were detected in a higher number of voxels when denoised simulated images were used for voxel-wise estimations, compared to quantification on raw simulated images. Variability of voxel-wise binding estimations on denoised clinical SPECT and PET images was also significantly diminished.

**Conclusion:**

In conclusion, noise removal from dynamic brain SPECT and PET images may optimize voxel-wise BP_ND_ estimations and detection of biological differences using SPM.

## Introduction

Molecular imaging using Positron Emission Tomography (PET) and Single Photon Emission Tomography (SPECT) is a powerful tool in the *in vivo* study of neuroreceptor systems in human and small-animal research. Quantification is most often performed on dynamic images (i.e. serial acquisitions of images with a very short duration) that permit the extraction of the temporal kinetic pattern of the radiotracer. Quantification is subsequently based on modeling of radiotracer kinetics. Quantitative analysis of dynamic PET and SPECT images is performed either at the regional level or at the voxel level: regional analysis of radiotracer kinetics implies an *a priori* definition of volumes-of-interest (VOI) in which radioactivity across voxels is averaged and examined as a whole. Alternatively, given the advances in the domain of instrumentation and image reconstruction, kinetic analysis may be performed on tissue-activity curves (TACs) from individual brain voxels to create binding parameter images that can be used for statistical analysis of differences at the voxel level [1–3].

Dynamic images, being composed of serial images of a very short duration, naturally suffer from high noise. This is a considerably limiting factor for the application of kinetic analysis at the voxel level, inducing bias and augmenting the variance of parameter estimates. In particularly noisy voxels, fitting the kinetic model may not be possible at all [1–3]. As a consequence, the statistical power to detect group differences in binding in the context of biological studies is seriously compromised and this is probably the main reason why VOI analysis still remains in the first line of neuroreceptor quantitative imaging. It is a serious impediment to the in-depth investigation of brain chemistry across the physiological and pathological spectrum. Indeed, subtle variations in neuroreceptor binding may be confined to cellular populations much smaller in size than the anatomically defined VOIs that do not necessarily correspond to the anatomical organization of neurotransmitter systems [4, 5] or the localization of other phenomena that affect the Central Nervous System (CNS), such as amyloid deposition [6], neuroinflammation [7] or epileptogenic foci [8]. VOI-wise analysis may confirm voxel-wise analysis or possibly underestimate biological differences [9, 10] and lead to type II statistical errors. In a similar way, functional and structural Magnetic Resonance Imaging (MRI) have had a major contribution in the understanding of brain function and pathology exactly because statistical inferences became possible at the voxel level.

The optimization of voxel-wise quantification in molecular imaging through noise reduction has been the aim of extensive research effort, the objective being to facilitate the application of kinetic models and the extraction of robust parameter estimates. Among these methods, factor analysis (FA) has shown its potential in denoising cardiological [11] and, more recently, small-animal brain imaging data [12, 13]. FA separates the signal of dynamic images into a finite number of factor-images [11, 14–16]. The rest of the signal is considered noise and discarded. Many other denoising approaches, have been developed over the years for denoising of PET and SPECT studies. Our group, for instance, has experience with wavelet denoising of brain PET studies [3]. Denoising of dynamic PET images has also been described using a non-local means denoising (NLM) approach [17], highly constrained backprojection (HYPR) [18], nonlinear spatio-temporal filtering [19] and context modelling using local neighborhood correlation [20]. Denoising techniques are extensively reviewed in [21, 22].

Given the well-established gains of denoising in terms of generating unbiased and precise estimates of binding parameters in PET and SPECT imaging, it is important to evaluate if noise removal increases the statistical power for the detection of group differences in voxel-wise parameter binding estimates with Statistical Parametric Mapping (SPM). A considerable number of studies have established the impact of noise [23–25] and processes related to noise, such as reconstruction [26, 27] on the detection of statistically significant differences at the voxel level. Wimberley et al. [28] evaluated the impact of denoising on voxel-wise calculations, but not with SPM, which is perhaps the most validated and certainly the most widely used approach in this domain [29]. In this study, we evaluate denoising of two GABA_A_-binding radiotracers, in [^123^I]iomazenil (IMZ) SPECT and [^11^C]flumazenil (FMZ) PET images in healthy human subjects. Using a simulation study of radiotracer binding augmentation, we determine the capacity of this approach to ameliorate the statistical power for the detection of biological differences. Importantly, kinetic analysis with and without FA is performed using two of the most widely used and best-established kinetic approaches, the Simplified Reference Tissue model (SRTM) [30, 31] using the basis function approach and Logan non-invasive graphical analysis [32]. We thus make the hypothesis that denoising optimizes the detection of significant differences in binding at the voxel level. Thus, a statistically significant difference in radiotracer binding between groups of scans that would otherwise remain undetected because of noise, is highlighted with SPM when denoising is applied.

## Materials and methods

### Subjects

Four male healthy subjects with ages ranging from 23 to 36 years (mean 27.4 ± 5.6) undertook one PET and one SPECT study each, with a two-to-three month interval between the scan sessions. All subjects gave their informed consent before scanning and the Research Ethics Committee of Geneva Hospital approved the study on the basis of cantonal and federal legislation in accordance with the Helsinki Declaration of 1975 (and as revised in 1983).

### PET and SPECT experiments

In this paper, we employed PET and SPECT images from a previous study of our group [33]. Acquisition and image reconstruction procedures have been described in detail elsewhere [33]. A whole body scanner (ADVANCE, GE, Medical System, Waukeska, WI) was employed for PET studies and the reconstruction of transaxial images was performed with a voxel size of 2.34 x 2.34 x 4.25 mm^3^. Photon attenuation was corrected with a 10-min transmission scan and the data were corrected for decay. The PET and SPECT scans employed in this paper consist of the first parts of a multi-injection procedure protocol [33]. For PET studies, an injection of [^11^C]FMZ (about 148 MBq) was followed by a 30-min scan of 17 frames of augmenting duration: 2 x 0.5 min; 10 x 1 min; 2 x 2 min; 3 x 5 min.

SPECT scan acquisitions were performed using a Toshiba GCA-9300A/HG triple-headed SPECT system in continuous rotation mode, using a super-high-resolution fan beam (SHR-FB) collimator. The triple-energy window method for scatter correction and the Chang filtered method of attenuation correction were applied as previously described [33]. Images were reconstructed with a final voxel size of 1.72 x 1.72 x 3.44 mm^3^. For the SPECT study, a single injection of 111 MBq of [^123^I]IMZ was used. A set of 25 sequential frames was collected over 170 min according to the following protocol: 5 x 2 min; 10 x 5 min; 10 x 11 min.

### Image processing

PET and SPECT images were processed using PMOD software (version 3.7, 2016, PMOD Technologies Ltd, Zurich, Switzerland). For anatomical localization of the cortical structures, a T1-weighted MR brain image volume was obtained for each subject (PICKER Eclipse 1.5T, TR = 15 ms, TE = 4 ms, pixel size, 0.98 x 0.98 x 1.10 mm^3^). Automatic co-registration of PET and SPECT images to the respective MRI images was performed using a normalized mutual information algorithm in the PMOD image fusion tool [34]. Motion correction was also applied on dynamic PET and SPECT images using the same tool.

Dynamic images were then processed in the Pixies software (Apteryx, Issy-les-Moulineaux, France) as previously described [12, 14–16, 35–38]. Briefly, FA is used for the decomposition of a series of dynamic images into a few elementary component-images. The decomposition is based on the distinct kinetic pattern of each component-image and is performed at the voxel level. Thus, the kinetic pattern of radioactivity in its voxel of the original (raw) dynamic image series, ${TAC}_{i}^{raw}(t)$, is expressed as a function of a finite number (k) of curves called factors f_k_, each one corresponding to a distinct radioactivity kinetic pattern and a set of factor-images α_k_ that represent the spatial distribution of the factors. Overall, the decomposition of the radioactive signal may be expressed using the following equation:

$${TAC}_{i}^{raw}(t)=\sum_{k=1}^{K}a_{k}(i)f_{k}(t)+e_{i}(t)$$ (1)

where e_i_(t) represents the error term for each voxel i at time t including both noise and modeling errors. In the present study, noise removal was the objective of FA, so all the component-images extracted from FA were examined together in one image and no individual component-image analyses was performed.

### Simulation study

**Fig 1:**
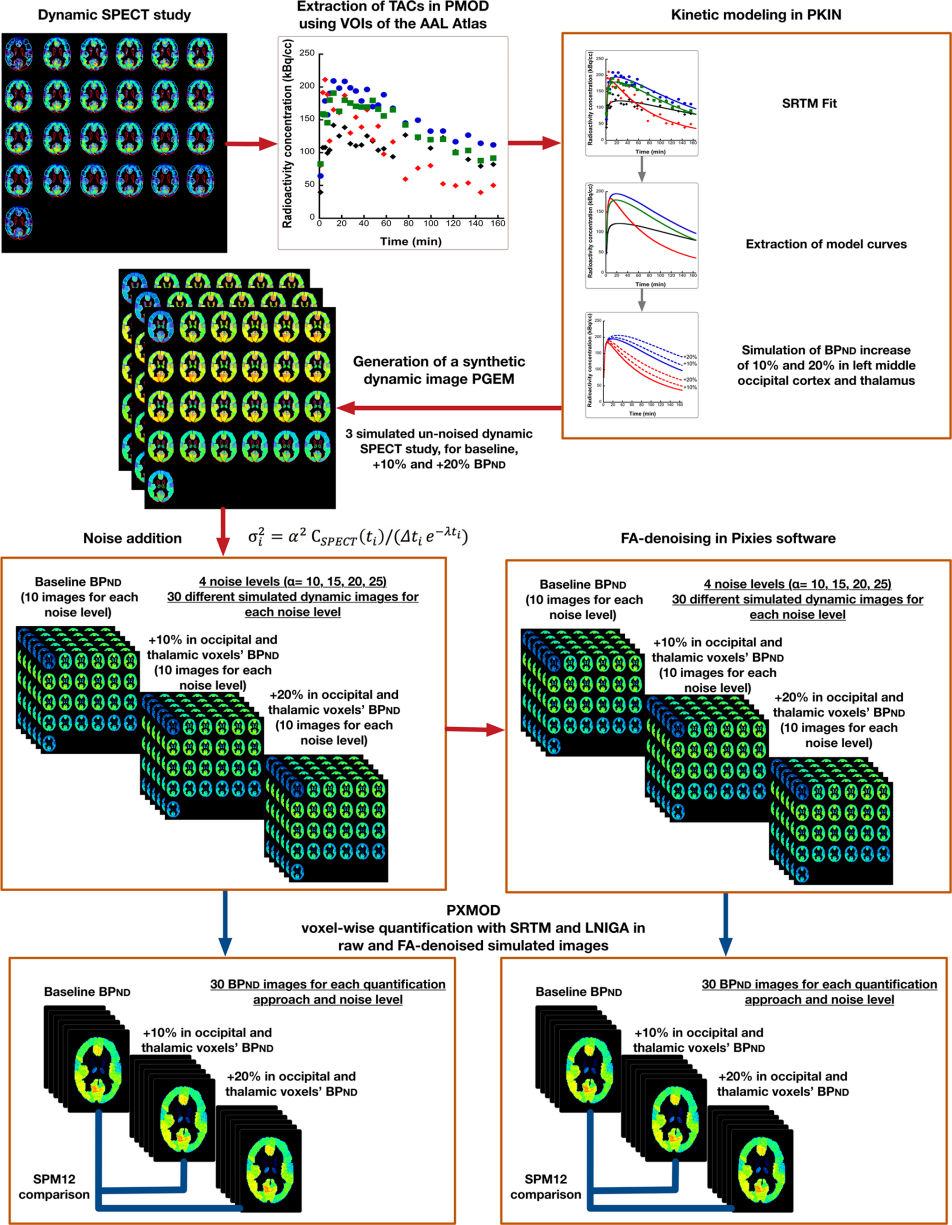
Schematic presentation of the simulation experiment.

A simulation study (schematically described in Fig 1) was designed to evaluate: 1) the potential of FA to denoise dynamic images, and 2) its impact in voxel-wise quantification and detection of biological group differences using SPM. Whole-brain dynamic SPECT synthetic volumes were generated using the PMOD Anatomy tool [39]. A set of VOIs defining the simulated images’ anatomy and a set of TACs defining the radioactivity kinetics in all the voxels of each corresponding VOI are required as input to the Anatomy tool. Pre-defined VOIs from the Automatic Anatomic Labeling (AAL) human brain atlas [40] included in PMOD were used. To simulate [^123^I]IMZ kinetics, TACs were extracted from one of the human dynamic SPECT studies using PMOD. These TACs were then fitted with SRTM in the PKIN tool [41] using the TAC from the pons VOI as a reference-region, as previously described [12], and model curves of this fit were extracted. The true simulated parameters are presented in S1 Table. The PKIN tool allows a simulation of a SRTM fit curve by modifying the binding potential (BP_ND_) parameter. So, we simulated model curves with a +10% and +20% difference in BP_ND_ compared to the original model curves in two of the brain VOIs, a high-binding (middle occipital cortex) and a low-binding region (thalamus). Three sets of model curves (baseline, +10% and +20%) were generated and subsequently employed in the Anatomy tool as the simulated images’ kinetics. The three un-noised simulated images thus represent human brain dynamic SPECT scans in which all voxels in a given VOI have identical kinetics, these of the input-curve that corresponds to this particular VOI.

Then, Gaussian noise was added [42–48] at four distinct levels *α* (10, 15, 20 and 25) with zero mean and variance $\sigma_{i}^{2}$:

$$\sigma_{v}^{2}={\alpha^{2}C}_{SPECT}(t_{v})/({\Delta t}_{v}e^{-\lambda t_{v}})$$ (2)

where λ is the decay constant λ = ln2/*T*_1/2_ and T_1/2_ is the half-life of the radiotracer (= 13.2 hours). C_SPECT_(*t*_*v*_) is the voxel activity corresponding to image frame *v*, *t*_*v*_ is the midtime and *Δt*_*v*_ is the duration of each frame. In order to perform statistical analysis of the simulated biological differences in radiotracer binding, ten synthetic dynamic images were simulated at each noise level for the baseline simulated image and these with the 10 and 20% augmentation in binding in the middle occipital cortex and the thalamus.

All dynamic images were processed with FA as described in the image processing section. Two prevalent factors (k = 2) were retained and the rest of the signal was considered as noise (images are thus designated as FA2c).

Voxel-wise kinetic analysis was performed in PXMOD [49] pixel-wise quantification tool in PMOD software, as previously described [12]. Two model configurations were used: the SRTM [30, 31] using the basis function method (hereon SRTM) and the Logan non-invasive graphical analysis [32] (hereon LNIGA) to generate parametric images of BP_ND_ [50]. For both models, PXMOD requires a preliminary fit of a VOI-extracted target-region TAC whose results serve as initial parameters for subsequent fitting of voxel TACs. Thus, a middle occipital cortex TAC was employed at this stage, along with a pons TAC that served as a reference-region for both models.

Bias compared to the simulated BP_ND_ value and coefficient of variance (CV) of the BP_ND_ estimations were the criteria for the evaluation of FA-denoising in parametric quantification. Estimated BP_ND_ values over the voxels of the high-binding region, the middle occipital cortical VOI (n = 2098) and the low-binding one, the thalamic VOI (n = 1057) were extracted from one simulated “subject”-simulated dynamic image of each noise and simulated BP_ND_ value (baseline, +10% and +20%).

Parametric images of the two groups of simulated increase in BP_ND_ (+10% and +20%) were compared to baseline simulated images with SPM. This comparison was performed for every noise level separately. The evaluation criterion in this case was the percentage of voxels in which the simulated statistically significant difference was highlighted with SPM (hereon, the recovery). SPM12 (Wellcome Trust Centre for Neuroimaging, UCL, London, UK), integrated in Matlab (R2016, Mathworks Inc, USA), was used. Comparison was performed by means of two-sample t-test with differences being considered significant at a p<0.001 with cluster size >100 voxels. This threshold was fixed after a preliminary series of SPM statistical comparisons between two independent groups of simulated images with no simulated difference in BP_ND_. These comparisons were performed for all levels of noise. A threshold of 10 voxels already eliminated all type I statistical errors arising from multiple comparisons, thus a threshold of 100 voxels was considered far more adequate [51]. No normalization or spatial smoothing was applied as all the simulated images were created on the basis of the same VOI template.

To verify that the SPM-highlighted voxels were not merely an artifact of a bias in radiotracer kinetics induced by FA and that retaining two factors is a valid configuration for FA that does not distort the original simulated raw signal, we plotted the averaged voxel-wise TACs from the middle occipital and thalamic regions of one simulated raw scan and the respective FA-corrected one. An analysis of the average percent normalized residuals between the true simulated and FA-denoised dynamic simulated images’ TACs was also performed [45]. For a given voxel i, the residual at time-point (t), is

$$R_{i}(t)={TAC}_{i}^{true}(t)-{TAC}_{i}^{FA2c}(t)$$ (3)

and the percent normalized residuals

$$=\frac{R_{i}(t)}{{TAC}_{i}^{true}(t)}\;x\;100$$ (4)

The study described above in this section simulated low and high noise levels to evaluate if denoising may benefit PET (low-noise) and SPECT (high-noise) imaging. However, IMZ and FMZ imaging described in this paper, employ quite distinct scanning protocols, with FMZ PET requiring a much shorter scan (30 min) compared to IMZ SPECT (170 min). We thus performed a secondary simulation study using TACs extracted from one of the subjects of the PET study, using the same approach as described for the SPECT simulation. A +10% difference in BP_ND_ values was simulated in the middle occipital cortex and the thalamus. 10 simulated brain volumes were generated for the baseline and the +10% BP_ND_ level. A Gaussian noise with α = 10 was added (given that PET studies have lower noise levels than SPECT). Denoising using FA was performed (two factors were retained). Voxel-wise quantification of BP_ND_ was performed with the SRTM and parametric images were analyzed using SPM, as described for the SPECT simulation study. The recovery (%) of voxels, in which a difference in BP_ND_ was simulated, was the outcome of this study.

### FA-denoising of human PET and SPECT data

This part of our study evaluated the effect of FA-denoising on the coefficient of variance of the BP_ND_ estimates at the voxel level in real human PET and SPECT data. The FA-denoised dynamic images from the four subjects were employed for quantification. Given that PXMOD tool does not provide the estimated parameters’ coefficient of variation, voxel-wise estimations were performed using PKIN tool. This procedure is computationally expensive and particularly time-consuming. Thus, the estimations were limited to one axial slice of the images (slice 40 of the AAL atlas, comprised of roughly 3500 voxels that correspond to brain parenchyma). TACs were fitted with the SRTM and the LNIGA in PKIN tool using the pons as reference-region and BP_ND_ and associated coefficients of variation were estimated. CV associated to the BP_ND_ values, estimated in the voxel level on this particular axial slice were averaged across the subjects and compared between raw and FA-denoised data by means of a two-sample t-test. In addition, parametric images (corresponding to axial slice 40) of BP_ND_ and CV were produced for visual comparison.

## Results

Table 1 presents the bias as percent difference from true simulated values of voxel-wise estimated BP_ND_ in the occipital and thalamic voxels of raw and FA-denoised simulated images, for all noise levels, along with percent CV. BP_ND_ estimated with the SRTM presents a bias ranging from -2.45 to 3.66% depending on the level of simulated noise. In FA-denoised images, the bias induced in average BP_ND_ estimates is higher, ranging from -10.66 to 7.63%. Regarding LNIGA, bias in BP_ND_ values was larger in raw data. Indeed, increased simulated noise resulted in an–almost- linear increase in bias in LNIGA estimates, ranging from -.4 to -22.9%. In FA-denoised simulated images, bias ranged from -4.82 to 3.88%. Variability of voxel-wise estimated BP_ND_ values was consistently lower in FA-denoised simulated images than in corresponding raw ones with the same level of simulated noise, for both kinetic models (Table 1).

**Table 1:** Percent bias and coefficient of variability of mean BP_ND_ in simulated occipital cortex and thalamus voxel-wise TACs as a function of simulated noise and application of FA.

		Occipital Cortex				Thalamus				Occipital Cortex (+10%)				Thalamus (+10%)				Occipital Cortex (+20%)				Thalamus (+20%)			
		Bias(%)		CV(%)		Bias(%)		CV(%)		Bias(%)		CV(%)		Bias(%)		CV(%)		Bias(%)		CV(%)		Bias(%)		CV(%)	
	Noise	RAW	FA2c	RAW	FA2c	RAW	FA2c	RAW	FA2c	RAW	FA2c	RAW	FA2c	RAW	FA2c	RAW	FA2c	RAW	FA2c	RAW	FA2c	RAW	FA2c	RAW	FA2c
**SRTM**	**10%**	0.39	6.46	5.26	2.76	2.44	-6.71	10.71	5.88	0.18	4.26	5.14	2.56	0.56	-8.89	11.05	6.10	0.16	2.45	4.74	2.40	1.02	-8.63	11.06	5.56
	**15%**	0.78	6.46	8.16	4.23	3.05	-7.32	17.16	10.53	-0.18	4.44	7.83	4.25	3.33	-8.33	16.67	10.30	-0.65	2.62	6.75	3.99	2.03	-9.14	15.92	9.50
	**20%**	0.98	7.05	10.27	6.22	5.49	-7.32	20.81	14.47	-0.18	4.62	9.25	5.60	3.33	-8.89	20.97	14.02	-1.47	2.95	8.31	4.93	1.02	-10.66	19.60	13.07
	**25%**	0.78	7.63	10.87	7.09	3.66	-8.54	22.35	14.67	-0.53	5.33	10.36	6.75	1.11	-10.6	21.43	15.53	-2.45	2.62	8.89	5.42	3.05	-8.63	20.69	13.89
**LNIGA**	**10%**	-2.40	3.14	4.36	3.58	-3.97	-1.32	7.59	6.04	-2.67	2.17	4.46	3.43	-5.42	-3.61	7.01	5.63	-3.04	1.22	4.55	3.46	-5.00	-2.78	7.02	5.71
	**15%**	-4.99	3.14	6.61	5.38	-9.27	-0.66	13.14	10.00	-5.68	2.17	6.90	5.56	-9.64	-2.41	12.00	9.88	-5.78	1.07	6.95	5.42	-10.00	-2.78	11.73	9.14
	**20%**	-8.69	3.33	9.11	7.51	-14.57	-0.66	17.05	13.33	-9.85	2.00	8.89	7.36	-16.3	-2.41	16.55	13.58	-9.44	1.37	9.08	7.51	-16.67	-3.89	15.33	12.72
	**25%**	-13.31	3.88	10.23	9.07	-21.19	-1.99	20.17	14.86	-14.5	2.84	10.74	9.58	-22.9	-4.82	19.53	15.19	-16.13	1.67	10.71	9.28	-20.00	-1.67	18.06	13.56

**Fig 2:**
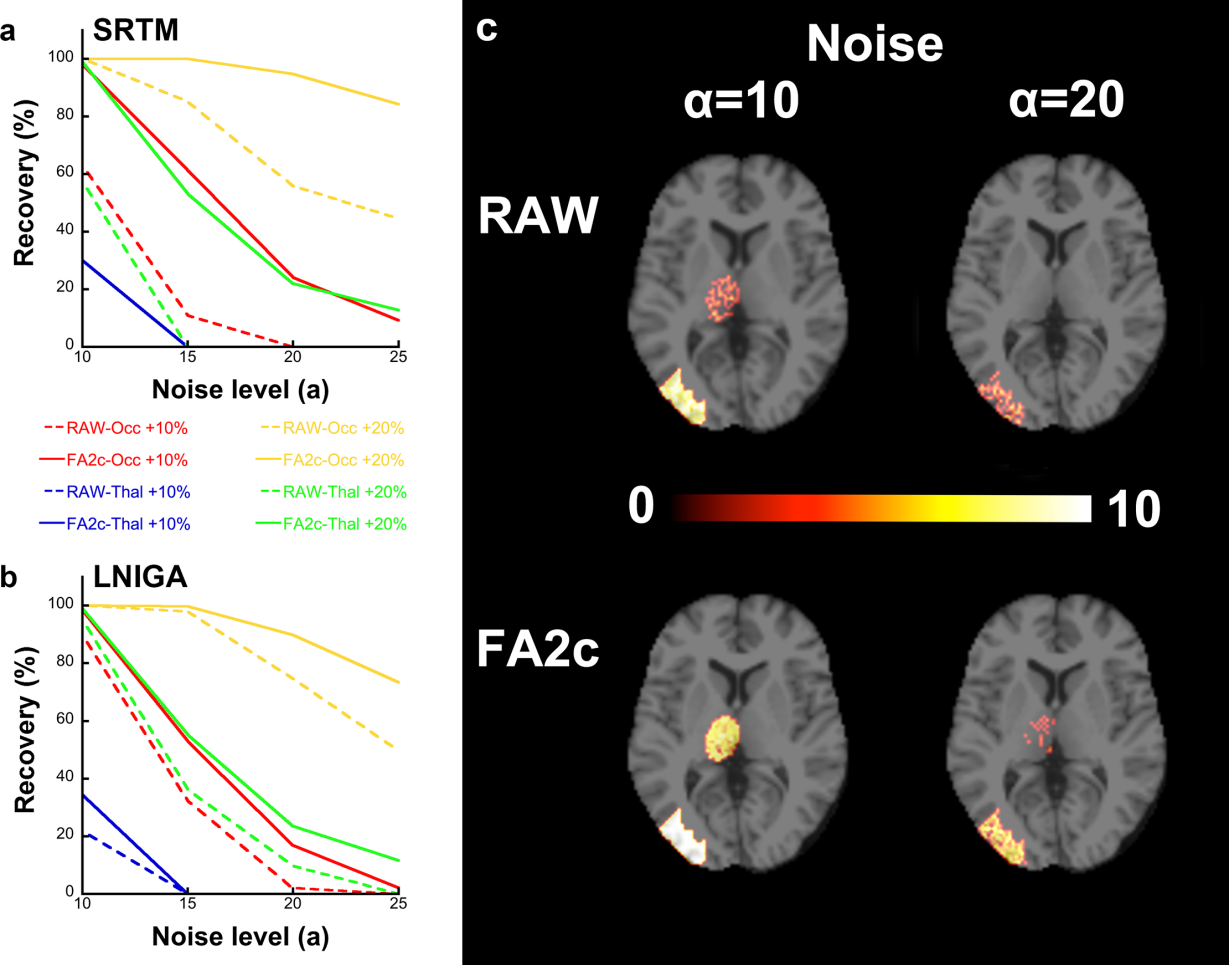
Recovery (%) (vertical axis) of voxels, in which a difference in radiotracer binding was simulated, plotted against the level of simulated noise (horizontal axis) and the simulated difference in simulated parameters between the groups of scans (represented by different colors). Continuous lines represent recovery from FA-denoised images while dashed lines represent raw-image derived recovery. Two subplots (a-b) correspond to the different quantification approaches. (c) An axial slice of a parametric image of T-values derived from SPM analysis of difference of binding of two groups of parametric images produced after voxel-wise application of SRTM for a low- (α = 10) and high- noise (α = 20) level as compared in raw and FA-denoised images. Note that FA-denoising leads not only to an increase in recovery of simulated voxels but in an increase in T-values associated with the recovered voxels.

Fig 2A and 2B presents the result of statistical analysis of difference in voxel-wise estimated BP_ND_ between 1) the group of simulated images with baseline values and 2) a simulated 10 or 20% increase in occipital and thalamic regions, using SPM. Recovery (%) is plotted against the level of simulated noise and the simulated difference in parameter values between the groups of images. In general, recovery was consistently higher for lower noise levels. Detection of difference in the occipital cortex (the high binding region) was consistently more efficient than in the thalamus (low binding VOI) for all simulated increases in the parameter values (10 and 20%) and simulated noise levels. Application of FA ameliorated recovery for both models (Fig 2A and 2B). The impact of FA2c was higher in SRTM than in LNIGA. Indeed, concerning SRTM results, an increase of up to 50% in recovery was observed, notably in the occipital voxels for both simulated levels of BP_ND_ while the increase in recovery in thalamic voxels was more evident for the 20% simulated increase in BP_ND_ (Fig 2A). On the other hand, FA-denoising of simulated images before quantification with LNIGA gave a roughly 20% higher recovery of voxels compared to raw ones (Fig 2B). SPM analysis of difference in voxel-wise estimates of BP_ND_ is not only associated with a higher number of voxels where significant differences are found between the two groups of simulated images but also with higher T-values as depicted in Fig 2C. This figure presents an axial slice of an SRTM-derived parametric image of T-values for a low- and high-noise level as compared in raw and FA-denoised images. Regarding the secondary simulation study using PET data, voxel recovery was higher in FA-denoised images, as was the case in the SPECT simulation study (0% in the middle occipital cortex and 21.5% in the thalamus in raw vs 63% in the middle occipital cortex and 50.9% in the thalamus in FA-denoised images).

**Fig 3:**
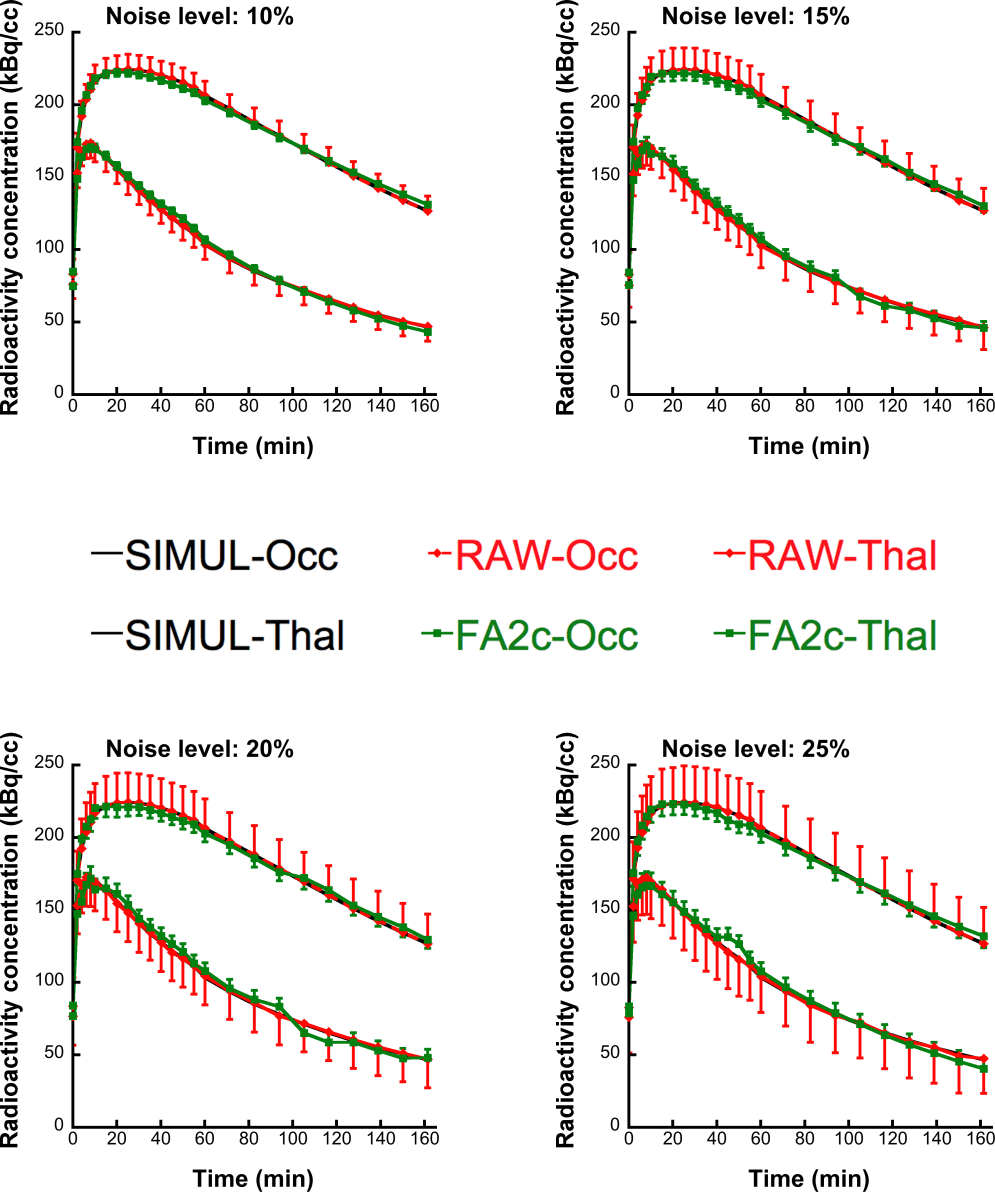
Average TACs over the voxels in the occipital and thalamic VOI, extracted from raw (RAW-Occ and RAW-Thal respectively) and FA-denoised (FA2c-Occ and FA2c-Thal, respectively) simulated images. The same TACs are shown for all four levels of noise simulation in comparison to corresponding TACs from the simulated, un-noised dynamic image (SIMUL-Occ and SIMUL-Thal). FA application does not induce any considerable bias in voxel-wise kinetics and markedly diminishes its variability.

**Fig 4:**
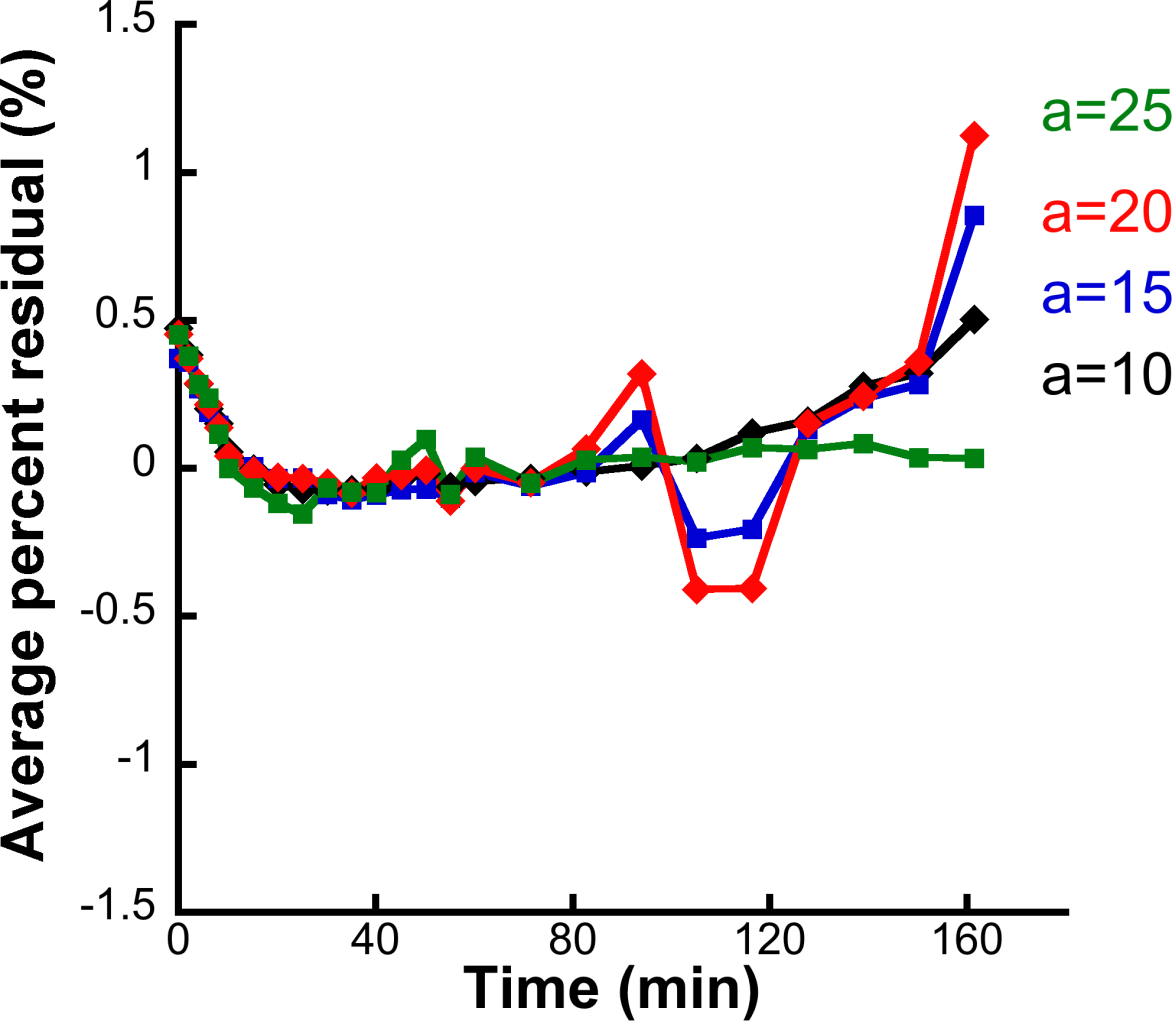
Average percent normalized residuals between the true simulated and FA-denoised dynamic simulated image TACs for all time-frames and for the four levels of noise (marked with different colors).

Fig 3 depicts the average TACs of the voxel-kinetics in the middle occipital and thalamic regions, extracted from raw and FA-denoised simulated images. The respective TACs are shown for all four levels of noise. Variability of radioactivity concentration in the VOIs was consistently lower in FA2c simulated images than in raw ones for all noise levels. SD values of radioactivity concentration are also shown in Fig 3. The average radioactivity kinetic profile in the VOIs is highly similar in raw and FA-denoised simulated images for all noise levels, suggesting that a minimal bias is introduced by the FA. This is further supported quantitatively by the analysis of percent normalized residuals between TACs from raw and FA-denoised simulated images that were close to zero for all four simulated noise levels (α = 10, 15, 20, 25) as shown in Fig 4.

**Fig 5:**
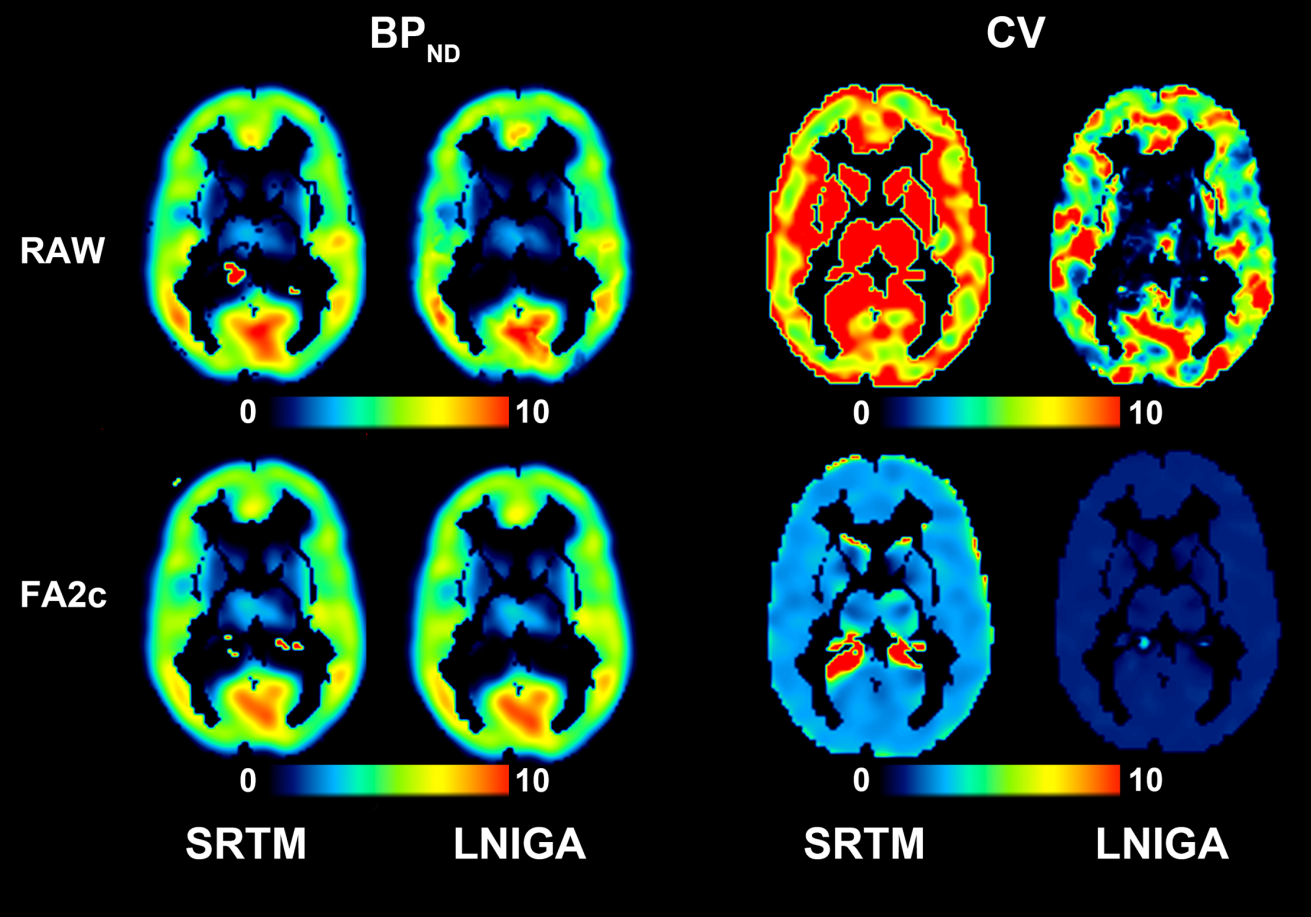
Parametric BP_ND_ (ml/ml) and CV (%) values of binding parameters obtained from voxel-wise quantification using SRTM and LNIGA on an axial slice on a [^123^I]IMZ SPECT image from one participant of the study. Denoising with FA gives equal, if not superior quality parametric images of BP_ND_ while markedly diminishes its variability.

**Fig 6:**
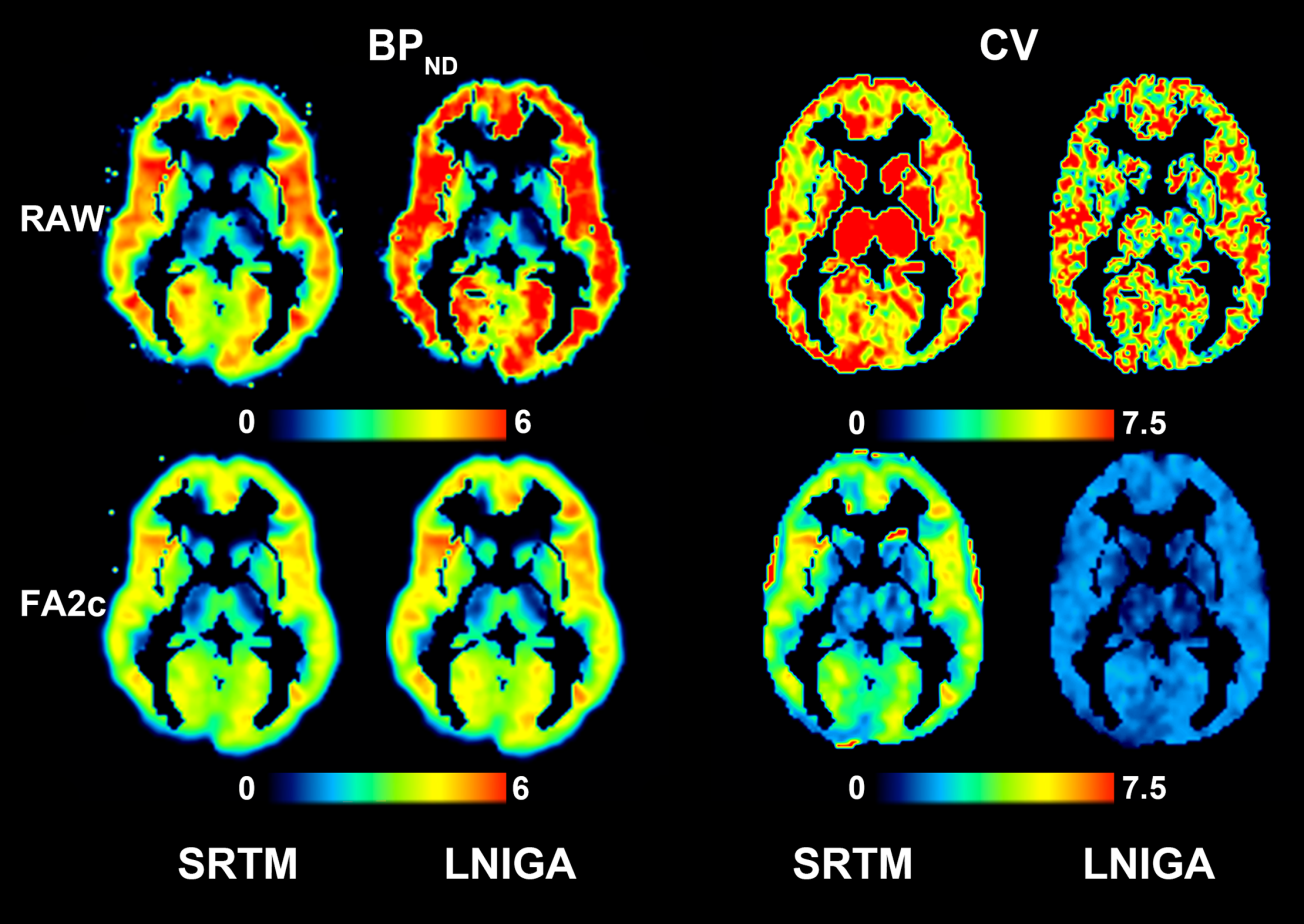
Parametric BP_ND_ (ml/ml) and CV values of binding parameters obtained from voxel-wise quantification using SRTM and LNIGA on an axial slice on a [^11^C]FMZ PET image from one participant of the study. As with SPECT images of Fig 5, denoising with FA gives equal, if not superior quality parametric images of BP_ND_ while markedly diminishes its variability.

Parametric estimations of BP_ND_ and their associated CV values on one axial slice from a real clinical scan are shown in Fig 5 for SPECT and Fig 6 for PET images. Upon visual inspection, FA2c application produces high-quality BP_ND_ images. The associated CV values are remarkably smaller when binding is estimated on denoised images with respect to estimations on raw images. For SRTM, average CV of BP_ND_ values estimated on raw data from four clinical scans was 15.10% versus 3.57% in FA-denoised data. Similarly, for LNIGA, average CV values were 7.59% and 1.39%. In PET data, average CV of SRTM-derived BP_ND_ values in raw data was 8.99% versus 3.26% in FA-denoised data. For LNIGA, respective average CV values were 8.57% and 2.47%. Differences in CV values of BP_ND_ estimations from raw and FA-denoised images were highly statistically significant (p<0.0001 for all differences).

## Discussion

In this study, we evaluated the impact of a denoising method on the statistical analysis of differences in radiotracer binding at the voxel level using SPM. Denoising methods are usually evaluated on the basis of their effects on the variability of BP_ND_. However, diminishing variability does not directly translate into more statistical power to detect differences at the voxel level by SPM, as any possible (differential) bias that may be induced by the denoising method is not taken into account. It is the reason for choosing to produce three-dimensional simulated image studies and not just a set of TACs. Such a study is not feasible unless there is an *a priori* knowledge of the true biological parameter values and group differences. For this reason, biological differences in binding in groups of simulated images were produced as a “ground truth”. Levels of noise that were added in dynamic simulated images in this simulation study were chosen to correspond to noise levels found in PET dynamic images (for α = 10 and 15) and highly noised SPECT images (α = 20 and 25). This was verified by comparing median CV of BP_ND_ values of SRTM applied in voxels from an axial slice of a real clinical SPECT and a PET image of this study with corresponding values from simulated images (data not shown).

Regarding binding parameter estimates, FA2c and raw image-associated bias differed considerably with respect to the kinetic model that was employed for quantification. BP_ND_ estimates from SRTM in raw simulated images were minimally biased compared to estimates from FA-denoised images. Biases from the application of FA in both series of simulated images were noise-independent. On the contrary, binding estimates resulting from LNIGA on raw simulated images presented the expected noise-dependent negative bias [1] that was minimal and much less noise-dependent in estimates from FA-denoised simulated images. Consistently across SRTM and graphical methods, variability of BP_ND_ estimates resulting from FA-denoised simulated images was lower than that from corresponding raw ones.

FA2c application on simulated dynamic images results in a considerable amelioration in terms of detection of a BP_ND_ difference in the voxel level for SRTM and LNIGA with this effect being particularly evident for high-binding voxels. Interestingly, the effect is more evident for estimates resulting from the application of SRTM than it is for results of graphical methods, perhaps due to the marked increase in precision of BP_ND_ estimates and in spite of the induction of bias. The basis function method SRTM [31] is already more resistant to noise than the original SRTM [30] and the results of this study demonstrate that further amelioration is possible if FA-denoised images are quantified with the basis function method SRTM. Quantifying raw simulated images with the SRTM was inferior to LNIGA in terms of voxel recovery. However, application of SRTM on FA-denoised simulated images proved not only superior to raw but also to denoised-image LNIGA estimates. Denoising with FA substantially diminishes the variability of voxel-wise dynamic radioactivity concentration, as demonstrated in Fig 3 without induction of any considerable systematic bias as TACs are highly similar for all simulated noise levels. Moreover, a quantitative analysis of the possible bias that FA could induce to the voxel kinetics confirmed that it is, indeed, negligible. Normalized residuals from the comparison of TACs from FA-denoised simulated images (Eqs 3 and 4) with the true simulated un-noised dynamic image take average values close to zero for all noise levels (Fig 4). This rules out that any distortion in radiotracer kinetics could underlie the increase in recovered voxels and that this increase in voxel recovery indeed results from the higher precision of FA-derived BP_ND_ estimates.

Beyond simulations, applying FA2c in real human SPECT and PET data demonstrated the highly significant effect of denoising on the precision of BP_ND_ estimation. While BP_ND_ estimated on raw images provides high quality images, the CV of these values is high, both in SPECT and PET images. With respect to this parameter, denoising with FA not only provided BP_ND_ images of excellent quality but also remarkably augmented the precision of these parameters, as shown in Figs 5 and 6.

The choice of the number of factors in FA lies on the kinetic properties of the [^123^I]IMZ and [^11^C]FMZ. In the present study, given the minimal non-specific component of the kinetics of these radiotracers [52], the retention of only two prevalent factors seems biologically justified and should correspond to the specific and free component of the radioactive signal. This argument is also supported by the resemblance of TACs from simulated average raw and FA-denoised simulated images (Fig 3) and the analysis of residuals from the comparison of TACs from FA-denoised simulated images (Eqs 3 and 4) with the true simulated un-noised dynamic image take average values close to zero for all noise levels. Moreover, preliminary evaluation of FA with retention of three and four prevalent factors gave results that were virtually indistinguishable from raw data in terms of recovery of voxel-wise differences in binding using SPM (data not shown).

The number of image-factors that are decomposed by FA correspond to biologically meaningful components of the radiotracer kinetics, even though the separate identification of the different factors may need more complex scanning protocols [14]. Nevertheless, this is not problematic in the context of a denoising study, in which the distinct image-factors are not examined separately but they are added together in one denoised image. Indeed, FA is a two-step procedure: in the first step, a principal component analysis (PCA) is performed [45]. In the second step, constraints are used to extract biologically meaningful image-factors. In the context of noise removal, only the first step is performed. Consequently, the results FA-denoising are essentially identical to the results of PCA.

The results of our study point to the potential for increase of statistical power of molecular neuroimaging studies with FA-denoising. FA is, to our point of view, a powerful tool in both SPECT and PET clinical imaging, as its positive impact was evident for the whole range of simulated noise levels, corresponding to low “PET-level” (α = 10) to high “SPECT-level” noise images (α = 25). Interestingly, recovery of voxels from FA2c-processed images with a high “SPECT-level” noise was comparable to the recovery from raw images with a low “PET-level” noise, particularly when SRTM was employed for quantification (Fig 2A). Furthermore, the secondary simulation study based on FMZ PET data demonstrated that the beneficial effect of FA-denoising was observed even with dynamic images with a duration as short as 30 min, thus further supporting the applicability of denoising in voxel-wise quantification. In the real clinical imaging data (Figs 5 and 6), FA significantly augmented the precision of parametric binding estimations on both SPECT and PET real clinical images. Denoised SPECT and PET images provided BP_ND_ values with nearly identical variability (in terms of CV). These findings suggest that 1) both SPECT and PET could benefit from FA and 2) extending the use of dynamic SPECT (that has the advantage of being cheaper and more available than PET) is feasible in clinical neuroimaging research with comparable statistical power to PET, provided that denoising is applied. In the present study, in terms of clinical utility, another potential application of FA could be in radioactive dose reduction that is necessary to minimize exposure of participants in longitudinal studies. Reducing dose is directly translated into augmented noise, an effect that could be corrected for by FA. Finally, in the case of radiotracers that have an inherently low binding in the brain (e.g. TSPO radiotracers) voxel-wise statistical analysis may be considerably optimized.

There are several limitations in our study. First, no comparison of FA with other denoising methods is performed. Indeed, the objective of this paper was to demonstrate the benefits of denoising on voxel-wise quantification and SPM analysis in molecular neuroimaging, not necessarily of FA in particular, which is employed here as an example of a denoising method. The quality of other denoising methods that have been recently described for human and small animal imaging [17, 18, 28, 45, 53, 54] is not challenged. Nevertheless, these methods have fundamental differences with each other and they may thus have a different impact on voxel-wise quantification. As a consequence, the results of this study may not be generalized to all denoising methods without prior validation. A second limitation is that we used the scans from a previous study of our group that were reconstructed using a filtered backprojection (FBP) method [33]. FBP has been largely replaced nowadays and noise characteristics may differ between the various reconstruction methods. Nevertheless, in all types of image reconstruction, the impact of noise on dynamic images and especially in voxel-wise quantification remains an important issue [17, 18, 28, 45, 53] and, in accordance with the conclusion of the present work, denoising dynamic images may enhance the statistical power of studies using SPM for voxel-wise quantification analysis. Another limitation is that the noise model was quite simple and added directly on TACs. Indeed, more complex noise models, using Monte Carlo simulations as well as using noise addition in the sinogram level should be more realistic [55, 56]. Overall, despite the simplicity of the employed methods, the present paper presents a straightforward message, that there is a need for further application of voxel-wise quantification in the study of brain physiology and pathology. The results of this paper constitute a demonstration of the power of denoising for this purpose.

FA is based on the recognition of kinetic patterns of a radioactivity signal and thus is only applicable to dynamic PET and SPECT studies. This means that static images may not be denoised with FA. However, static images inherently have lower noise levels than the short-duration frames of dynamic studies, maybe having no need for extra denoising. This does not compromise the applicability of the proposed method. Indeed, dynamic scan protocols in molecular neuroimaging are widely employed, particularly in the context of research neurotransmission studies.

## Conclusion

In the current study, denoising of dynamic [^123^I]IMZ and [^11^C]FMZ images is employed. A simulation study using [^123^I]IMZ dynamic images demonstrates that denoising ameliorates the extraction of voxel-wise differences in radiotracer binding, detected using SPM, when parametric quantification is performed with SRTM and LNIGA. Thus, dynamic image denoising could, after appropriate validation for other radiotracers, considerably optimize clinical studies and detection of biologically meaningful differences in the voxel level.

## Supporting information

S1 Table True SPECT simulated parameter values. (PDF)

S2 Table True PET simulated parameter values. (PDF)
