# Supplementary material for: Dynamic image denoising for voxel-wise quantification with Statistical Parametric Mapping in molecular neuroimaging
Source: PLoS One. 2018 Sep 5;13(9):e0203589. doi: 10.1371/journal.pone.0203589 (PMC6124809; doi:10.1371/journal.pone.0203589)
Supplement: S1 Table — (PDF) [file pone.0203589.s001.pdf]

S1 Table True SPECT simulated parameter values.

VOI	BP_{ND}	R_1	k_2	k_2'	k_{2a}
**Precentral_l**	2.9510	0.9530	0.0380	0.0399	0.0096
**Precentral_r**	2.7941	0.8835	0.0342	0.0387	0.0090
**Frontal_Sup_l**	3.2027	0.9611	0.0375	0.0390	0.0089
**Frontal_Sup_r**	3.3146	0.9802	0.0366	0.0374	0.0085
**Frontal_Sup_Or**	4.0800	0.8953	0.0285	0.0318	0.0056
**Frontal_Sup_Or**	3.8082	0.8652	0.0289	0.0334	0.0060
**Frontal_Mid_l**	3.3162	0.9852	0.0383	0.0389	0.0089
**Frontal_Mid_r**	3.8649	1.0655	0.0369	0.0347	0.0076
**Frontal_Mid_Orl**	3.0533	0.6365	0.0228	0.0358	0.0056
**Frontal_Mid_Orl**	7.7891	0.8138	0.0170	0.0209	0.0019
**Frontal_Inf_Ope**	3.5759	0.8951	0.0408	0.0456	0.0089
**Frontal_Inf_Ope**	5.5820	1.3222	0.0335	0.0254	0.0051
**Frontal_Inf_Tri_**	3.1798	0.8699	0.0347	0.0399	0.0083
**Frontal_Inf_Tri_**	3.9281	0.9612	0.0309	0.0321	0.0063
**Frontal_Inf_Orb**	4.1756	0.9046	0.0351	0.0388	0.0068
**Frontal_Inf_Orb**	4.4963	0.9482	0.0282	0.0298	0.0051
**Rolandic_Oper_**	4.5279	1.2150	0.0365	0.0300	0.0066
**Rolandic_Oper_**	4.4827	1.1754	0.0427	0.0364	0.0078
**Supp_Motor_Ar**	3.2220	1.0526	0.0404	0.0384	0.0096
**Supp_Motor_Ar**	3.2951	1.0623	0.0419	0.0395	0.0098
**Olfactory_l**	3.5314	1.2156	0.0481	0.0396	0.0106
**Olfactory_r**	3.5889	1.3775	0.0391	0.0284	0.0085
**Frontal_Sup_Me**	4.0373	1.1000	0.0463	0.0421	0.0092
**Frontal_Sup_Me**	3.7265	0.9781	0.0420	0.0429	0.0089
**Frontal_Med_Or**	6.3464	1.3430	0.0393	0.0293	0.0054
**Frontal_Med_Or**	5.3243	1.1587	0.0478	0.0413	0.0076
**Rectus_l**	5.3336	1.2844	0.0417	0.0325	0.0066
**Rectus_r**	5.6258	1.2727	0.0420	0.0330	0.0063
**Insula_l**	5.6162	1.3551	0.0485	0.0358	0.0073
**Insula_r**	6.0049	1.3526	0.0463	0.0342	0.0066
**Cingulum_Ant_l**	5.9628	1.4034	0.0492	0.0351	0.0071
**Cingulum_Ant_r**	7.0483	1.5390	0.0367	0.0238	0.0046
**Cingulum_Mid_l**	6.2055	1.4523	0.0456	0.0314	0.0063
**Cingulum_Mid_r**	6.8813	1.7291	0.0509	0.0294	0.0065
**Cingulum_Post_**	4.8652	0.9902	0.0512	0.0517	0.0087
**Cingulum_Post_**	5.8682	1.0712	0.0372	0.0347	0.0054
**Hippocampus_l**	1.1680	0.7781	0.0251	0.0322	0.0116
**Hippocampus_r**	1.6853	0.8109	0.0158	0.0194	0.0059
**Parahippocamp**	2.3938	0.8322	0.0376	0.0452	0.0111
**Parahippocamp**	2.8674	0.9327	0.0284	0.0304	0.0073
**Amygdala_l**	2.5330	1.0021	0.0295	0.0295	0.0084
**Amygdala_r**	2.1141	0.8464	0.0212	0.0251	0.0068
**Calcarine_l**	5.9381	1.2721	0.0487	0.0383	0.0070
**Calcarine_r**	6.6826	1.3897	0.0474	0.0341	0.0062

**Cuneus_l**	4.8517	1.2326	0.0439	0.0356	0.0075
**Cuneus_r**	5.0173	1.2928	0.0495	0.0383	0.0082
**Lingual_l**	5.4399	1.1802	0.0441	0.0373	0.0068
**Lingual_r**	7.5090	1.3817	0.0456	0.0330	0.0054
**Occipital_Sup_l**	2.8692	0.7748	0.0380	0.0490	0.0098
**Occipital_Sup_r**	3.9750	1.0101	0.0435	0.0431	0.0087
**Occipital_Mid_l**	3.7066	1.0389	0.0395	0.0380	0.0084
**Occipital_Mid_r**	5.1139	1.1285	0.0462	0.0409	0.0076
**Occipital_Inf_l**	4.9147	1.0640	0.0368	0.0346	0.0062
**Occipital_Inf_r**	5.1667	1.0398	0.0375	0.0361	0.0061
**Fusiform_l**	4.3139	0.9641	0.0342	0.0355	0.0064
**Fusiform_r**	4.7032	1.1086	0.0323	0.0291	0.0057
**Postcentral_l**	2.7228	0.9092	0.0315	0.0347	0.0085
**Postcentral_r**	2.6480	0.8564	0.0332	0.0387	0.0091
**Parietal_Sup_l**	1.4217	0.6011	0.0299	0.0497	0.0123
**Parietal_Sup_r**	0.9473	0.3930	0.0258	0.0657	0.0133
**Parietal_Inf_l**	3.2101	1.0447	0.0396	0.0379	0.0094
**Parietal_Inf_r**	3.3379	0.9417	0.0371	0.0394	0.0086
**Supra_Marginal**	3.7154	0.9697	0.0411	0.0424	0.0087
**Supra_Marginal**	4.8363	1.1007	0.0419	0.0381	0.0072
**Angular_l**	3.4516	1.0057	0.0454	0.0451	0.0102
**Angular_r**	4.9716	1.2481	0.0399	0.0320	0.0067
**Precuneus_l**	3.6610	0.9081	0.0432	0.0476	0.0093
**Precuneus_r**	4.9536	1.1476	0.0389	0.0339	0.0065
**Paracentral_Lol**	1.6694	0.6278	0.0284	0.0453	0.0107
**Paracentral_Lol**	1.8866	0.8022	0.0342	0.0426	0.0118
**Caudate_l**	0.7759	1.0162	0.0128	0.0126	0.0072
**Caudate_r**	0.4053	0.9421	0.0324	0.0344	0.0231
**Putamen_l**	3.3429	1.5276	0.0451	0.0295	0.0104
**Putamen_r**	2.7763	1.3843	0.0422	0.0305	0.0112
**Pallidum_l**	1.7040	1.4670	0.0722	0.0492	0.0267
**Pallidum_r**	1.7044	1.2982	0.0369	0.0284	0.0136
**Thalamus_l**	1.3211	1.1030	0.0337	0.0305	0.0145
**Thalamus_r**	1.6395	1.0425	0.0267	0.0256	0.0101
**Heschl_l**	6.9118	1.4578	0.0546	0.0375	0.0069
**Heschl_r**	6.7089	1.6450	0.0564	0.0343	0.0073
**Temporal_Sup_**	4.7097	1.0769	0.0398	0.0369	0.0070
**Temporal_Sup_**	5.4152	1.2530	0.0391	0.0312	0.0061
**Temporal_Pole_**	2.4114	0.7388	0.0242	0.0328	0.0071
**Temporal_Pole_**	3.2301	0.8180	0.0215	0.0263	0.0051
**Temporal_Mid_l**	4.3106	0.9553	0.0349	0.0365	0.0066
**Temporal_Mid_**	6.3097	1.2140	0.0364	0.0300	0.0050
**Temporal_Pole_**	2.8611	0.6090	0.0204	0.0334	0.0053
**Temporal_Pole_**	2.4098	0.6320	0.0190	0.0300	0.0056
**Temporal_Inf_l**	4.1574	0.9525	0.0349	0.0367	0.0068
**Temporal_Inf_r**	5.4863	1.0524	0.0299	0.0284	0.0046

**Cerebellum_Cru**	4.5984	0.9661	0.0336	0.0347	0.0060
**Cerebellum_Cru**	4.1541	0.9493	0.0333	0.0351	0.0065
**Cerebellum_Cru**	3.0367	0.9277	0.0297	0.0320	0.0074
**Cerebellum_Cru**	2.2863	0.8462	0.0254	0.0300	0.0077
**Cerebellum3_l**	8.3692	1.0799	0.0205	0.0190	0.0022
**Cerebellum3_r**	3.1195	0.8291	0.0502	0.0605	0.0122
**Cerebellum45_l**	6.8526	1.3478	0.0398	0.0296	0.0051
**Cerebellum45_r**	5.4611	1.0506	0.0538	0.0512	0.0083
**Cerebellum6_l**	6.7318	1.2875	0.0468	0.0364	0.0061
**Cerebellum6_r**	7.3267	1.5131	0.0450	0.0297	0.0054
**Cerebellum7_l**	3.3696	1.1369	0.0398	0.0350	0.0091
**Cerebellum7_r**	3.0311	1.0989	0.0356	0.0324	0.0088
**Cerebellum8_l**	4.7739	1.3749	0.0446	0.0325	0.0077
**Cerebellum8_r**	3.5368	1.2486	0.0433	0.0347	0.0096
**Cerebellum9_l**	2.9397	1.2169	0.0325	0.0267	0.0083
**Cerebellum9_r**	3.2045	1.1683	0.0412	0.0353	0.0098
**Cerebellum10_l**	2.4772	0.5434	0.0280	0.0515	0.0081
**Cerebellum10_r**	2.7746	0.7180	0.0179	0.0249	0.0047
**Vermis12**	3.0698	0.9551	0.0456	0.0478	0.0112
**Vermis3**	2.5131	1.0085	0.0257	0.0255	0.0073
**Vermis45**	7.2659	1.2325	0.0279	0.0226	0.0034
**Vermis6**	0.0274	0.0428	9.8461	0.0039	0.0039
**Vermis7**	0.0341	0.0465	6.2264	0.0064	0.0064
**Vermis8**	0.0205	0.0356	6.8489	0.0045	0.0045
**Vermis9**	0.0247	0.0297	4.7721	0.0051	0.0051
**Vermis10**	0.0452	0.0358	2.8540	0.0093	0.0093
