# Supplementary material for: Dynamic image denoising for voxel-wise quantification with Statistical Parametric Mapping in molecular neuroimaging
Source: PLoS One. 2018 Sep 5;13(9):e0203589. doi: 10.1371/journal.pone.0203589 (PMC6124809; doi:10.1371/journal.pone.0203589)
Supplement: S2 Table — (PDF) [file pone.0203589.s002.pdf]

S2 Table True PET simulated parameter values.

VOI	BP_{ND}	R_1	k_2	k_2'	k_{2a}
**Precentral_**	1.9932	1.0152	0.4028	0.3967	0.1346
**Precentral_**	1.7445	0.9496	0.3609	0.3801	0.1315
**Frontal_Su**	1.9330	0.9651	0.3805	0.3942	0.1297
**Frontal_Su**	1.9600	0.9815	0.3902	0.3976	0.1318
**Frontal_Su**	0.9985	0.7047	0.2142	0.3039	0.1072
**Frontal_Su**	0.6038	0.5945	0.1598	0.2688	0.0997
**Frontal_Mi**	2.2622	1.1055	0.3972	0.3593	0.1218
**Frontal_Mi**	1.9413	0.9807	0.3781	0.3856	0.1286
**Frontal_Mi**	0.5864	0.6234	0.1735	0.2784	0.1094
**Frontal_Mi**	0.0000	0.4798	0.0847	0.1765	0.0847
**Frontal_Inf**	1.9997	1.0076	0.3696	0.3668	0.1232
**Frontal_Inf**	1.6879	0.9126	0.3525	0.3862	0.1311
**Frontal_Inf**	1.9477	0.9763	0.3447	0.3530	0.1169
**Frontal_Inf**	1.2346	0.7449	0.2935	0.3941	0.1314
**Frontal_Inf**	1.4469	0.8734	0.2727	0.3122	0.1114
**Frontal_Inf**	0.7618	0.6308	0.2082	0.3300	0.1181
**Rolandic_**	3.0027	1.3213	0.4496	0.3403	0.1123
**Rolandic_**	2.4302	1.1140	0.3907	0.3507	0.1139
**Supp_Moto**	2.3124	1.1154	0.4332	0.3884	0.1308
**Supp_Moto**	2.0999	1.1047	0.4203	0.3804	0.1356
**Olfactory_**	2.6950	1.1880	0.3438	0.2894	0.0930
**Olfactory_**	2.5712	1.2048	0.2995	0.2486	0.0839
**Frontal_Su**	2.5573	1.1586	0.4257	0.3674	0.1197
**Frontal_Su**	2.2257	1.0581	0.3915	0.3700	0.1214
**Frontal_Me**	2.3006	1.0181	0.3368	0.3308	0.1020
**Frontal_Me**	2.4469	1.1215	0.3679	0.3280	0.1067
**Rectus_l**	1.5480	0.9210	0.2478	0.2690	0.0972
**Rectus_r**	1.6180	0.9351	0.2572	0.2751	0.0983
**Insula_l**	3.1285	1.3247	0.4724	0.3566	0.1144
**Insula_r**	2.6830	1.1894	0.4198	0.3529	0.1140
**Cingulum_**	2.6161	1.1847	0.3885	0.3280	0.1074
**Cingulum_**	2.0398	0.9835	0.3683	0.3745	0.1212
**Cingulum_**	2.4387	1.2086	0.4641	0.3840	0.1350
**Cingulum_**	2.0607	1.0893	0.3980	0.3653	0.1300
**Cingulum_**	1.7077	1.0623	0.3955	0.3723	0.1460
**Cingulum_**	1.3023	0.9581	0.3422	0.3572	0.1486
**Hippocamp**	2.0236	0.9670	0.3440	0.3557	0.1138
**Hippocamp**	2.3844	1.0073	0.3671	0.3645	0.1085
**Parahippoc**	2.2982	1.0130	0.3398	0.3354	0.1030
**Parahippoc**	1.8910	0.9092	0.2725	0.2997	0.0942
**Amygdala_**	1.9843	0.8953	0.3317	0.3704	0.1111
**Amygdala_**	2.0417	0.9749	0.3209	0.3291	0.1055
**Calcarine_**	2.9812	1.2712	0.4604	0.3622	0.1157
**Calcarine_**	3.2652	1.3123	0.4802	0.3659	0.1126
**Cuneus_l**	2.6947	1.1990	0.4601	0.3838	0.1245
**Cuneus_r**	2.9353	1.2895	0.4795	0.3719	0.1219

**Lingual_l**	2.8359	1.1906	0.4368	0.3669	0.1139
**Lingual_r**	2.4597	1.1229	0.3926	0.3496	0.1135
**Occipital_S**	2.0435	1.0071	0.3772	0.3746	0.1239
**Occipital_S**	2.1988	1.0445	0.3866	0.3702	0.1209
**Occipital_M**	2.0650	0.9707	0.3655	0.3765	0.1192
**Occipital_M**	2.3296	1.0113	0.3871	0.3828	0.1163
**Occipital_I**	1.6534	0.8402	0.2935	0.3493	0.1106
**Occipital_I**	1.5340	0.7975	0.2811	0.3525	0.1109
**Fusiform_l**	2.3573	1.0676	0.3432	0.3215	0.1022
**Fusiform_r**	1.9004	0.9628	0.3500	0.3636	0.1207
**Postcentra**	1.8369	0.9906	0.3799	0.3835	0.1339
**Postcentra**	1.6615	0.9021	0.3396	0.3764	0.1276
**Parietal_Su**	1.7613	0.9255	0.3424	0.3700	0.1240
**Parietal_Su**	1.7631	0.8706	0.3315	0.3808	0.1200
**Parietal_In**	2.4350	1.1328	0.4050	0.3575	0.1179
**Parietal_In**	2.4312	1.0844	0.4241	0.3911	0.1236
**Supra_Mar**	2.2208	1.0792	0.3915	0.3628	0.1216
**Supra_Mar**	2.1982	1.0871	0.3874	0.3563	0.1211
**Angular_l**	2.5321	1.1870	0.4303	0.3625	0.1218
**Angular_r**	2.2966	1.1042	0.4248	0.3847	0.1289
**Precuneus**	2.4702	1.1468	0.4501	0.3925	0.1297
**Precuneus**	2.6816	1.2457	0.4489	0.3604	0.1219
**Paracentra**	1.5875	0.9373	0.3646	0.3890	0.1409
**Paracentra**	1.7748	1.0244	0.3548	0.3464	0.1279
**Caudate_l**	1.2585	1.0760	0.5097	0.4737	0.2257
**Caudate_r**	1.2582	1.1143	0.5042	0.4525	0.2233
**Putamen_l**	2.1478	1.3132	0.5052	0.3847	0.1605
**Putamen_r**	1.8821	1.2704	0.4786	0.3767	0.1661
**Pallidum_l**	1.8749	1.3033	0.4508	0.3459	0.1568
**Pallidum_r**	1.7678	1.1608	0.4146	0.3572	0.1498
**Thalamus_**	1.1237	1.1628	0.6362	0.5472	0.2996
**Thalamus_**	1.2142	1.1550	0.6875	0.5952	0.3105
**Heschl_l**	2.5900	1.2828	0.4795	0.3738	0.1336
**Heschl_r**	2.1432	1.0422	0.4409	0.4230	0.1403
**Temporal_**	2.2273	1.0006	0.3789	0.3787	0.1174
**Temporal_**	2.0346	0.9711	0.3476	0.3579	0.1145
**Temporal_**	1.6063	0.8314	0.2615	0.3146	0.1003
**Temporal_**	1.0890	0.6627	0.2116	0.3193	0.1013
**Temporal_**	1.7896	0.8790	0.3002	0.3415	0.1076
**Temporal_**	1.6148	0.8336	0.3047	0.3655	0.1165
**Temporal_**	0.6569	0.6153	0.1548	0.2516	0.0934
**Temporal_**	0.2942	0.4975	0.1207	0.2426	0.0932
**Temporal_**	1.0750	0.6561	0.2345	0.3574	0.1130
**Temporal_**	0.5810	0.5386	0.1701	0.3159	0.1076
**Cerebellum**	0.9583	0.8407	0.3247	0.3863	0.1658
**Cerebellum**	1.2110	0.9685	0.4248	0.4386	0.1921
**Cerebellum**	0.5121	0.6684	0.2384	0.3566	0.1577
**Cerebellum**	0.3469	0.5664	0.2309	0.4076	0.1714

**Cerebellum**	0.8554	0.8594	0.3985	0.4637	0.2148
**Cerebellum**	0.4585	0.8308	0.5653	0.6804	0.3876
**Cerebellum**	1.7356	1.0005	0.4094	0.4092	0.1496
**Cerebellum**	1.2858	1.0567	0.3622	0.3427	0.1584
**Cerebellum**	2.3649	1.3080	0.5193	0.3971	0.1543
**Cerebellum**	1.6992	1.2211	0.4759	0.3897	0.1763
**Cerebellum**	0.5815	0.6685	0.2489	0.3722	0.1574
**Cerebellum**	0.2111	0.4787	0.1675	0.3498	0.1383
**Cerebellum**	1.0104	0.8906	0.3372	0.3787	0.1678
**Cerebellum**	0.7221	0.7481	0.2225	0.2974	0.1292
**Cerebellum**	1.3865	0.9813	0.4074	0.4152	0.1707
**Cerebellum**	1.1834	0.9375	0.4119	0.4393	0.1886
**Cerebellum**	1.0777	0.9647	0.2802	0.2904	0.1348
**Cerebellum**	0.6554	0.7273	0.1974	0.2714	0.1192
**Vermis12**	0.2802	0.7668	0.5618	0.7326	0.4388
**Vermis3**	1.2352	1.0653	0.4169	0.3914	0.1865
**Vermis45**	1.8645	1.1871	0.4168	0.3511	0.1455
**Vermis6**	2.0143	1.1934	0.5193	0.4351	0.1723
**Vermis7**	1.8631	1.3191	0.5223	0.3960	0.1824
**Vermis8**	1.5157	1.2656	0.4605	0.3638	0.1830
**Vermis9**	1.8186	1.3279	0.5557	0.4184	0.1971
**Vermis10**	1.4821	1.2202	0.5272	0.4321	0.2124
